# Development and Application of Sub-Mitochondrial Targeted Ca^2 +^ Biosensors

**DOI:** 10.3389/fncel.2019.00449

**Published:** 2019-10-04

**Authors:** Markus Waldeck-Weiermair, Benjamin Gottschalk, Corina T. Madreiter-Sokolowski, Jeta Ramadani-Muja, Gabriela Ziomek, Christiane Klec, Sandra Burgstaller, Helmut Bischof, Maria R. Depaoli, Emrah Eroglu, Roland Malli, Wolfgang F. Graier

**Affiliations:** ^1^Molecular Biology and Biochemistry, Gottfried Schatz Research Center, Medical University of Graz, Graz, Austria; ^2^Energy Metabolism Laboratory, Institute of Translational Medicine, D-HEST, Swiss Federal Institute of Technology (ETH), Zurich, Switzerland; ^3^Department of Internal Medicine, Division of Oncology, Medical University of Graz, Graz, Austria; ^4^Department of Medicine, Brigham and Women’s Hospital, Harvard Medical School, Boston, MA, United States; ^5^BioTechMed-Graz, Graz, Austria

**Keywords:** Ca^2+^ measurements, biosensors, mitochondrial Ca^2+^, mitochondrial cristae, Ca^2+^, live cell imaging, mitochondria

## Abstract

Mitochondrial Ca^2+^ uptake into the mitochondrial matrix is a well-established mechanism. However, the sub-organellar Ca^2+^ kinetics remain elusive. In the present work we identified novel site-specific targeting sequences for the intermembrane space (IMS) and the cristae lumen (CL). We used these novel targeting peptides to develop green- and red- Ca^2+^ biosensors targeted to the IMS and to the CL. Based on their distinctive spectral properties, and comparable sensitivities these novel constructs were suitable to visualize Ca^2+^-levels in various (sub) compartments in a multi-chromatic manner. Functional studies that applied these new biosensors revealed that knockdown of MCU and EMRE yielded elevated Ca^2+^ levels inside the CL but not the IMS in response to IP_3_-generating agonists. Knockdown of VDAC1, however, strongly impeded the transfer of Ca^2+^ through the OMM while the cytosolic Ca^2+^ signal remained unchanged. The novel sub-mitochondrially targeted Ca^2+^ biosensors proved to be suitable for Ca^2+^ imaging with high spatial and temporal resolution in a multi-chromatic manner allowing simultaneous measurements. These informative biosensors will facilitate efforts to dissect the complex sub-mitochondrial Ca^2+^ signaling under (patho)physiological conditions.

## Introduction

**FIGURE 1:**
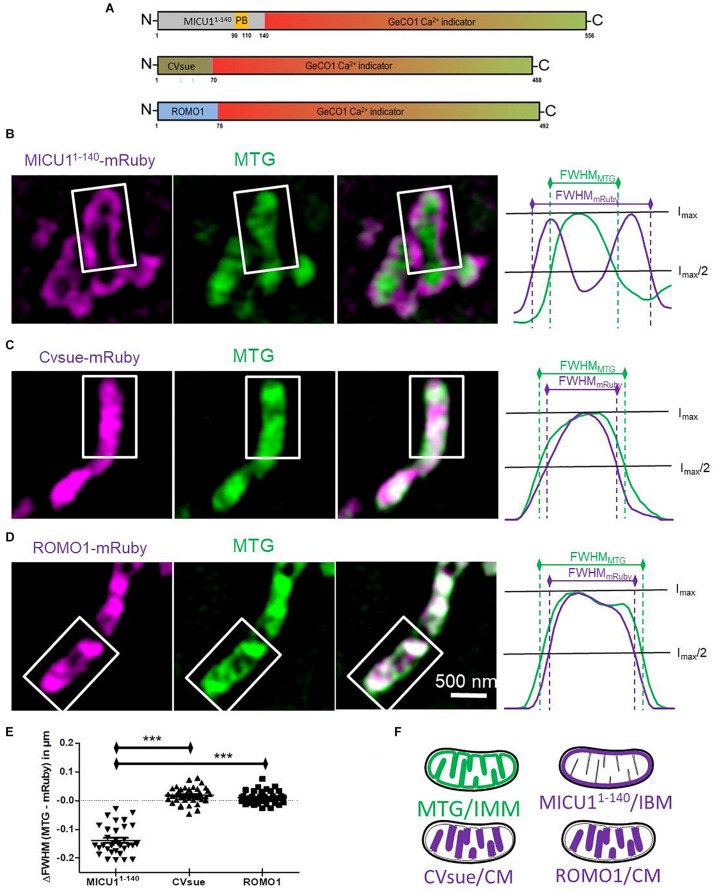
Differential sub-mitochondrial localization of MICU1^1– 140^-, CVsue- and ROMO1-mRuby. **(A)** Illustration of construct design of sub-mitochondrial targeting sequences fused with GeCO1 Ca^2+^ indicators. MICU1^1– 140^ including polybasic domain (PB), full length complex V subunit e and full length mutated reactive oxygen modulator 1 (ROMO1) were C-terminally tagged with red and green GeCO1 Ca^2+^ indicators. Numbers indicates amino acid positions. **(B–D)** Representative dual-color structural illumination microscopy (SIM) images of HeLa mitochondria stained with inner mitochondrial membrane-(IMM-) label MitoTracker Green (MTG, green, middle images) and expressing mRuby2 (magenta, left images) C-terminally tagged with either MICU1^1– 140^ **(B)**, CVsue **(C)** or ROMO1 **(D)**. Merged images show co-localization of green MTG and magenta mRuby fluorescence (right images). Sum of line scans within the representative rectangle areas were performed for the green and red fluorescence (right panels). Overlay of normalized fluorescence intensity curves indicates distances in full width at half maximum (FWHM) of sub-mitochondrial targeted mRuby (magenta curves) vs. MTG (green curves). **(E)** Calculation of differences in FWHM_*MTG*_ to FWHM_*mRuby*_ was performed by subtracting the distance given by the magenta curve from the green one. Statistical evaluation of ΔFWHM values calculated per mitochondrion; ^∗∗∗^*P* < 0.001. Images were obtained from 5 cells in each of 8 independent experiments on 4 different days (*n* = 8) for each targeting. **(F)** Schemes illustrating sub-mitochondrial localization given by the distinct targeting sequences.

Embedded between the outer (OMM) and the inner mitochondrial membrane (IMM) the mitochondrial intermembrane space (IMS) including the cristae lumen (CL) represents the smallest mitochondrial sub-compartment. This specialized area is of upmost importance for the regulation of mitochondrial protein import, lipid homeostasis, energy metabolism and metal ion exchange to maintain cellular functions (Frey and Mannella, 2000; Herrmann, 2010; Shoshan-Barmatz et al., 2018). While the OMM is permeable for molecules below 5 kDa via VDAC1 (Camara et al., 2017), the IMM is impermeable. Thus, the transfer of biomolecules across the IMM into the mitochondrial matrix relies on transporters, carriers and exchangers. Among these the mitochondrial Ca^2+^ uptake machinery displays a frequently studied mechanism due to its importance for the regulation of various mitochondrial functions. The molecular nature of the mitochondrial Ca^2+^ uniporter is a complex formation of several interacting proteins located in the IMM including the pore forming subunit mitochondrial Ca^2+^ uniporter (MCU) (Baughman et al., 2011), the essential mitochondrial regulator (EMRE) (Sancak et al., 2013) and the two gatekeeper opponents, the mitochondrial Ca^2+^ uptake 1 and 2 (MICU1 and MICU2) (Kamer and Mootha, 2014). Recently, we and others demonstrated that MICU1 forms multimers and rearranges its oligomeric state as soon as Ca^2+^ enters by binding to the EF hand motifs that are localized toward the IMS (Wang et al., 2014; Waldeck-Weiermair et al., 2015), which in turn regulates MCU/EMRE channel activity. Notably in most non-excitable cells the endoplasmic reticulum (ER) represents the main source of Ca^2+^ to be requested into the mitochondrial matrix. The direct transfer of Ca^2+^ from the ER into the mitochondrial matrix has been studied intensively and was described to be take place in focal contact sites between the two organelles, the mitochondria-associated membranes (MAMs) (van Vliet et al., 2014). The formation of MAMs and as a consequence the transfer of Ca^2+^ from the ER to the mitochondria is crucially dependent on the distance between the two organelles (Szymański et al., 2017). On the other hand, the voltage dependent anion channel 1 (VDAC1) represents the most abundant porin in the OMM accounting for its permeability for cations like Ca^2+^ (Camara et al., 2017). As one of the most studied mitochondrial proteins VDAC1 does not just serve as a non-selective hole, but contributes to numerous Ca^2+^ dependent functions such as Ca^2+^-induced apoptosis (Weisthal et al., 2014), Ca^2+^-modulated energy production (Cárdenas et al., 2010; Shoshan-Barmatz and Mizrachi, 2012), global Ca^2+^ signaling (Berridge et al., 2003) and ER-to-mitochondria Ca^2+^ transport (Szabadkai et al., 2006; Gautier et al., 2016). For the latter it has been suggested that VDAC1 may form supra-molecular complexes that occur at MAMs by the interaction with GRP75 (Szabadkai et al., 2006), mitofusin-2 (Shoshan-Barmatz et al., 2018) and the IP_3_ receptor 1 (Szabadkai et al., 2006). Indeed, regulation of mitochondrial Ca^2+^ uptake is especially critical in neurons. On the one hand excessive mitochondrial Ca^2+^ induce cell death by the release of pro-apoptotic proteins like cytochrome C that is mainly harbored within mitochondrial cristae (Cogliati et al., 2016) leading to neurodegenerative pathologies like Parkinson’s disease (PD), Alzheimer’s disease (AD), Huntington’s disease (HD), amyotrophic lateral sclerosis (ALS) (Britti et al., 2018). Although these diseases are commonly based on mitochondrial Ca^2+^ dysfunction, the progression of each depends on its specific molecular mechanism. Accordingly, dysregulation of VDAC1 has been described to induce both, PD and AD, either by altered dopamine homeostasis (Alberio et al., 2014) or mediating amyloid β toxicity (Smilansky et al., 2015). In HD it has been reported that the capacity of Ca^2+^ buffering is impaired (Gellerich et al., 2008) and in ALS it is hypothesized that chronic excitotoxicity induce permanent ER Ca^2+^ depletion leading to mitochondrial Ca^2+^ overload (Grosskreutz et al., 2010). On the other hand regulated mitochondrial Ca^2+^ uptake is a prerequisite for neuronal function and survival (Rugarli and Langer, 2012). Aside from stimulating ATP production, transient increases in mitochondrial Ca^2+^ particularly occur at synapses and regulates neurotransmitter release, synaptic transmission and excitability (Pivovarova and Andrews, 2010) by rapidly clearing Ca^2+^ from the cytosol (MacAskill et al., 2010). For instance, in a recent work it has been demonstrated by simultaneously detecting cytosolic and mitochondrial Ca^2 +^ using targeted genetically encoded indicators, namely cytosolic G-GeCO1 and mitochondrial R-GeCO1, that Ca^2+^ is strongly biased toward mitochondria in axon terminals in zebrafish neurons (Mandal et al., 2018). However, since mitochondrial Ca^2+^ buffering is tightly shaping the spatiotemporal profiles of intracellular Ca^2+^ we herein sought to fill the “missing link” of genetically encoded Ca^2+^ probes between the cytosol and the mitochondrial matrix. In order to develop such tools that allow sub-mitochondrial recordings of respective Ca^2+^ signals as prerequisite in a more detailed investigation of the sub-mitochondrial Ca^2+^ signaling and homeostasis, in the present work we focused on the targeting of already widely used genetically encoded Ca^2+^ sensors (i.e., GeCO1-like Ca^2+^ indicators) to the IMS or the mitochondrial cristae. We identified specific targeting sequences for distinct sub-mitochondrial protein targeting and fused them with single FP-based various GeCO1-like Ca^2+^ indicators (Figure 1A) that even allow simultaneous recordings of the Ca^2+^ signals in two (sub-) compartments and enabled us to take a closer look in the kinetics of Ca^2+^ transfer from the ER to mitochondria.

## Materials and Methods

### Materials and Buffers

Chemicals and reagents for solutions and buffers were purchased from Carl Roth (Karlsruhe, Germany). Histamine, monensin, and BHQ were obtained from Sigma-Aldrich (Vienna, Austria). Nigericin was from Tocris (Abingdon, United Kingdom) and ionomycin from Abcam Biochemicals (Cambridge, United Kingdom). For storage cells were maintained in a buffer (storage buffer) containing (in mM): 2 CaCl_2_ 135 NaCl, 5 KCl, 1 MgCl_2_, 1 HEPES, 2.6 NaHCO_3_, 0.44 KH_2_PO_4_, 0.34 Na_2_HPO_4_, 10 D-glucose, 0.1% vitamins, 0.2% essential amino acids, and 1% penicillin/streptomycin pH adjusted with NaOH to 7.4 prior to imaging experiments. Therefore, cells were perfused in a physiological HEPES-buffered solution (2CaNa) composed of (in mM): 2 CaCl_2_ 135 NaCl, 5 KCl, 1 MgCl_2_, 1 HEPES, 10 D-glucose, pH adjusted with NaOH to 7.4 and stimulated with either BHQ or histamine in either a Ca^2+^ free solution or a nominal Ca^2+^ free solution containing EGTA instead of 2 CaCl_2_.

### Cell Culture and Transfection

In this study we exclusively used a HeLa cell model that was cultivated at passage >50 on low fluorescence glass cover slips with a diameter of either 15 or 30 mm in DMEM (Sigma-Aldrich, Vienna, Austria) containing 10% FCS (PAA, Pasching, Austria), penicillin (100 U/ml) and streptomycin (100 U/ml) in a humidified incubator (37°C, 5% CO_2_/95% air). Cells were transfected in a serum-free medium using 3 μg/ml TransFast^TM^ Transfection reagent (Promega, Madison, WI, United States) together with 1–2 μg of plasmid(s) and/or 100 nM of the respective siRNA(s) per ml 2 days before experiments.

### Plasmid and siRNA Material

The plasmid encoding mutated ROMO1 at positions 15, 27, and 42 from potential ROS modulating cysteines to serines (ROMO1) was synthesized by General Biosystems Inc., (Morrisville, NC, United States) and the vectors encoding for GEMGeCO1, CARGeCO1, and mt-CARGeCO1 were purchased from Addgene (Cambridge, MA, United States) and used as vector templates for the N-terminal insertion via *Xho*I/*Bam*HI of either MICU1^1–140^, CVsue or ROMO1 using the primers as follows: MICU1 forward 5′-CCCTCTAGACTCGAG CATG TTTCGTCTGAACTCACT-3′; MICU1 position 420 reverse 5′- GGTTGGATCC TTCAAGGTGGCAAAATATCGG-3′; CVsue forward 5′-AAACTCGAG CATGGTGCCACCGGTGCAGGT- 3′; CVsue position 206 reverse 5′-CCCGGATCC TTTAATAT GCTGTCATCTTCTGCC-3′; ROMO1 forward 5′-AAATCTAG ACTCGAGCATGCCGGTGGCCGTGGGT-3′ and ROMO1 position 255 reverse 5′-CATAGGATCC CGGATGCCCATCCCA ATGG-3′. For SIM these targeting sequences were subcloned in frame with a C-terminal red fluorescent protein (mRuby). All siRNAs were purchased from Microsynth (Balgach, Switzerland) and their (sense strands, 5′-3′) sequences were: ACACUAGGCACCGAGAUUA (siVDAC1) for silencing hVDAC1 (Arif et al., 2019); GCCAGAGACAGACAAUACU (hMCU-si1) and GGAAAGGGAGCUUAUUGAA (hMCU-si2) for silencing hMCU; GAACUUUGCUGCUCUACUU for silencing hEMRE and UUCUCCGAACGUGUCACGU as a scrambled Control siRNA. It should be noted that the siRNAs for the knockdown of hMCU and hEMRE had been already verified and used before in the present HeLa cell type (Waldeck-Weiermair et al., 2015; Gottschalk et al., 2019) and thus a verification of the efficiency due to the siRNA mediated knockdown was only performed for VDAC1 siRNA in this work (Supplementary Figure S6).

### Quantitative RT-PCR

Total cellular RNA was isolated from HeLa cells transfected with either siRNA against VDAC1 or the scrambled Control siRNA with the PEQLAB total RNA isolation kit (Peqlab, Erlangen, Germany), followed by reverse transcription to cDNA, performed in a thermal cycler (Peqlab) using the high-capacity cDNA reverse transcription kit (Applied Biosystems, Foster City, CA, United States). The qPCR reaction was set up with the GoTag^®^ qPCR Master Mix (Promega, Mannheim, Germany) together with gene-specific primers (Invitrogen, Vienna, Austria). Experiments were performed on a LightCycler 480 (Roche Diagnostics, Vienna, Austria). Relative expression of specific genes was normalized to human GAPDH, as a housekeeping gene. Primer sequences were as follows: VDAC1 for: 5′-GGACTGAGTACGGCCTGACGTT-3′, VDAC1 rev: 5′-CAGTCCACGTGCAAGCTGATCT-3′, GAPDH (QuantiTect^®^ Primer Assay Hs_GAPDH, Qiagen, Hilden, Germany).

### Ca^2+^ Imaging Experiments

Ca^2+^ imaging was performed on a digital wide field microscope, the iMIC (Till photonics, Gräfelfing, Germany) equipped with a 40× objective (alpha Plan Fluar 40×, Zeiss, Göttingen, Germany) and an ultrafast switching monochromator, the Polychrome V (Till Photonics). Illumination of GEM-GeCO1 targeted sensors was performed at 430 nm excitation and emissions were collected with a dichrotome dual emission filter set (dichroic 535dcxr, CFP emitter 482/18 nm, and YFP emitter 535/3 nm). pH calibration was performed as described previously (Burgstaller et al., 2019) in a Ca^2+^-free or Ca^2+^ containing buffer supplemented with 10 μM ionomycin each to establish Ca^2+^ unbound and Ca^2+^ bound states of targeted GEMGeCO1. CARGeCO1 based Ca^2+^ indicators were excited at 575 nm and emitted at 600 nm. For simultaneous measurements, GEM- and CARGeCO1 targeted sensors were alternately excited for 400 ms each at 430 and 575 nm. Emissions derived from both sensors were taken in 3 s interval. Alternatively, we used an ultrafast switching mode where both Ca^2+^ indicators were excited for 150 ms in a 310 ms interval. During the measurements cells were continuously perfused by using a gravity-based perfusion system (NGFI, Graz, Austria) and images were recorded with a charged-coupled device (CCD) camera (AVT Stingray F-145B, Allied Vision Technologies, Stadtroda, Germany). Data acquisition and control of the digital fluorescence microscope was performed using the live acquisition software version 2.0.0.12 (Till Photonics).

### Super-Resolution Structured Illumination Microscopy (SIM)

Structured Illumination Microscopy experiments were performed as described previously (Gottschalk et al., 2018). In brief, transfected HeLa cells either expressing MICU1^1–140^-mRuby, CVsue-mRuby or ROMO1-mRuby were loaded with 0.5 μM MitoTracker^TM^ Green (MTG, Thermo Fisher Scientific, Waltham, MA United States) in storage buffer for 40 min, once washed and imaged in 2CaNa with a super-resolution CFI SR Apochromat TIRF 100x-oil (NA 1.49) objective mounted on a Nikon-Structured Illumination Microscopy (N-SIM^®^) System with standard wide field and SIM filter sets and equipped with two Andor iXon3^®^ EMCCD camera mounted to a Two Camera Imaging Adapter (Nikon Austria, Vienna, Austria). Co-localization of MTG fluorescence intensities with MICU1^1–140^-mRuby, CVsue-mRuby and ROMO1-mRuby in the CM and IBM were measured via line scan analysis within a defined rectangle area. Normalized sum of intensities profiles derived from green (MTG) and red (mRuby) fluorescence were calculated to define the full width at half maximum (FWHM) as a measurement for the label latitude. MTG-staining was used as an internal reference for the IMM to determine the distance variation of sub-mitochondrial targeted mRuby at basis of the FWHM.

### Statistical Analysis of Data

Statistical analysis for SIM experiments were performed using one way ANOVA and Bonferroni’s *post hoc* test. The acquired data of Ca^2+^ measurements were analyzed by the GraphPad Prism software version 5.01 (GraphPad Software, San Diego, CA, United States). Data are presented as mean ± standard error of mean (SEM) of independent experiments (*n*) throughout the whole manuscript. For comparisons between two groups, two-tailed Student *t*-test was used for evaluation of statistical significance and a *P* value between 0.01 and 0.05 (*p* Student’s *t*-test) was considered significant and indicated with “^∗^”, *P* between 0.001 and 0.01 as very significant with “^∗∗^,” and *P* < 0.001 as highly significant with “^∗∗∗^.” For comparisons across multiple groups, one-way ANOVA with Bartlett’s test for equal variances and Bonferroni’s Multiple Comparison test were used for evaluating statistical significance expressed as described above.

## Results

### Development of Sub-Mitochondrial Targeted Ca^2+^ Biosensors

We and others have predicted that the mitochondrial calcium uptake 1 protein (MICU1) is a specific IMS protein via its N-terminal targeting sequence (Petrungaro et al., 2015; Waldeck-Weiermair et al., 2015). Accordingly, we generated a potentially IMS-targeted mRuby variant using the first 140 amino acids that includes the polybasic region of MICU1, but none of the two EF-hand motifs or the multimerization sites. HeLa cells expressing MICU1^1–140^-mRuby were subsequently loaded with the IMM specific dye MitoTracker^TM^ Green (MTG). Super-resolution structure illumination microscopy (SIM) revealed that MICU1^1–140^-mRuby was exclusively directed to the inner boundary membrane (IBM) (Figures 1B,E and Supplementary Figure S1A). For targeting the sensor into the CL, we utilized a recently identified protein referred to as reactive oxygen species modulator 1 (ROMO1) that acts especially in the CL (Norton et al., 2014; Cogliati et al., 2016). Moreover, we used the subunit e of complex V (CVsue) that has been already shown to target within the CL (Rieger et al., 2014; Figures 1C,E and Supplementary Figure S1A) to structurally and functionally verify the targeting of ROMO1. In its size ROMO1 is comparable with CVsue and is also anchored with only one transmembrane domain into the cristae membrane. To disable ROMO1’s function as ROS modulator while preserving its feature as a targeting peptide we mutated all four ROS sensitive cysteine residues to rather ROS-inert serine residues at the amino acid positions 15, 27, 42, and 79. Fusion of ROMO1 to the N-terminus of an mRuby yielded predominantly CL localization that was comparable to that of CVsue targeting as revealed via SIM (Figures 1D,E and Supplementary Figure S1A). SIM data were randomly recorded under all conditions and line plot analyses were performed showing no influence of ROMO1-, CVsue- or MICU1^1–140^-mRuby expression on mitochondrial thickness (Supplementary Figures S1B,C). Using these newly identified targeting sequences MICU1^1–140^, CVsue, and ROMO1 we generated differentially targeted green and red GeCO1 biosensors aiming to visualize Ca^2+^ within the IMS or CL compartments (Figures 1A,F).

### Testing the Functionality of the Sub-Mitochondrially Targeted Ca^2+^ Biosensors

**FIGURE 2:**
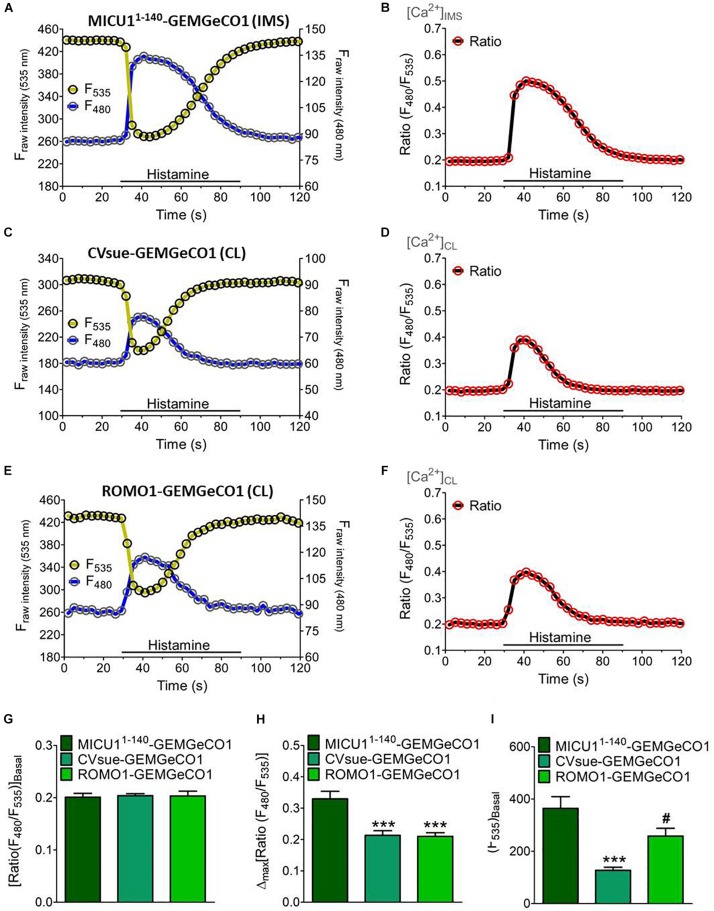
Dynamics of sub-mitochondrial targeted GeCO1-like Ca^2+^ indicators. Single fluorescence intensities over time of MICU1^1– 140^-GEMGeCO1 **(A)**, CVsue-GEMGeCO1 **(C)** and ROMO1-GEMGeCO1 **(E)** in HeLa cells excited at 430 and 480 nm (blue curve) and 535 nm (yellow curve) emission upon stimulation with 100 μM histamine in a nominal Ca^2+^ free buffer. Calculated ratio (green curves) from the respective left panels by the division of F_480_ by F_535_, representing Ca^2+^ concentration levels ([Ca^2+^]) in the IMS **(B)** and the CL **(D,F)**. **(G–I)** Statistical analysis of HeLa cells expressing either MICU1^1– 140^-GEMGeCO1 (dark green, *n* = 17), CVsue-GEMGeCO1 (aqua green, *n* = 16) or ROMO1-GEM-GeCO1 (light green, *n* = 15) and evaluation of ratiometric signals at basal levels **(G)** in response to histamine treatment **(H)** and intensiometric basal intensities at 535 nm reflecting sensor expression levels **(I)**. ^∗∗∗^*P* < 0.001 of CVsue-GEMGeCO1 and ROMO1-GEMGeCO1 vs. MICU1^1– 140^-GEMGeCO1 and ^#^*P* < 0.05 of ROMO1-GEMGeCO1 vs. CVsue-GEMGeCO1.

We generated two groups of differentially targeted Ca^2+^ biosensors including the ratiometric GEMGeCO1 (Zhao et al., 2011) or intensiometric CARGeCO1 (Wu et al., 2013; Supplementary Table S1). For imaging of the ratiometric GEMGeCO1 we used a standard CFP/YFP emission filter. Recording the single fluorescence intensities of the localized sensor constantly revealed a 5-fold higher signals in the YFP channel compared to the CFP emission at basal Ca^2+^ levels (Figures 2A,C,E). Analyses of all three sub-mitochondrial constructs resulted in comparable ratio signals under resting conditions (Figures 2B,D,F,G). However, the signals upon IP_3_-mediated Ca^2+^ mobilization by histamine (100 μM) was more than 50% higher by using the IMS specific MICU1^1–140^-GEMGeGO1 in comparison to both CL targeted probes (Figure 2H). Although the two Ca^2+^ indicators within the CL, CVsue-GEMGeCO1 and ROMO1-GEMGeCO1, performed almost identical, the expression level of the ROMO1 targeted probe was significantly higher enabling a better resolution of signals (Figure 2I). Moreover, testing pH sensitivity of MICU1^1–140^ or ROMO1 localized GEMGeCO1 revealed relative pH insensitivity of the probes at neutral and higher pH as previously reported for the recombinant GEMGeCO1 protein (Zhao et al., 2011; Supplementary Figure S2). We also constructed the corresponding sub-mitochondrial targeted intensiometric red CARGeCO1 variant which displays a low fluorescent signal under resting conditions and greatly lights up in the presence of Ca^2+^. According to the low expression level together with the low fluorescence intensity of a CVsue-CARGeCO1 indicator we assumed that this probe is hardly detectable at basal Ca^2+^ levels and thus, visualization of CL localized CAR-GeCO1 was only performed by using the ROMO1 targeting sequence. In line with the results obtained by the ratiometric GEMGeCO1, statistical analyses of IMS or CL targeted CARGeCO1 derived signals resulted in comparable histamine responses indicating to distinct sub-mitochondrial Ca^2+^ concentrations upon Ca^2+^ mobilization with an IP_3_ generating agonist (Supplementary Figure S3).

### Multi-Chromatic Imaging of Sub-Mitochondrial Ca^2+^ Responses

**FIGURE 3:**
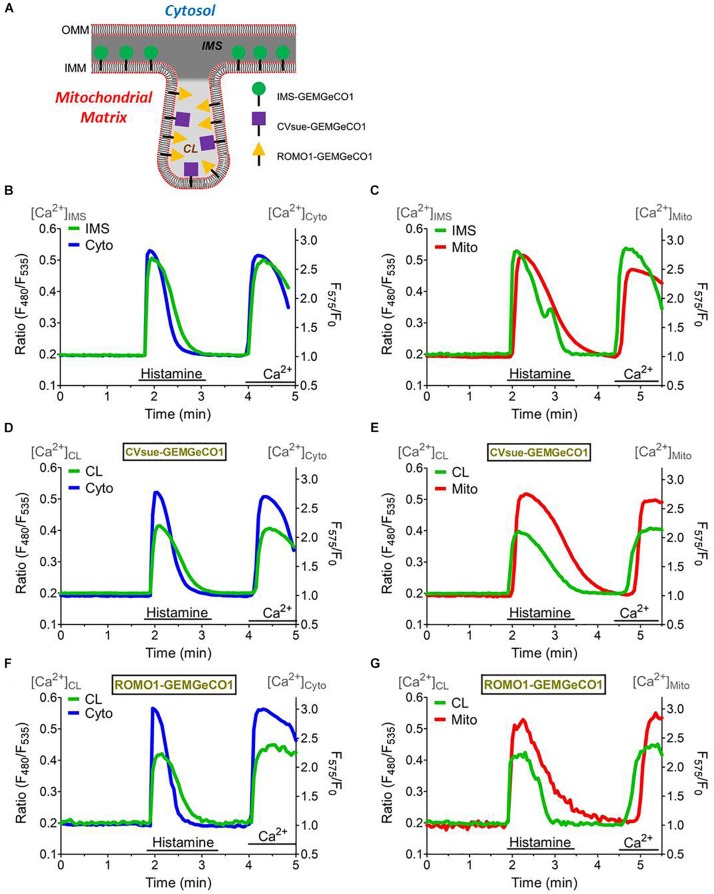
Simultaneous measurements of the sub-mitochondrial Ca^2+^ sensors with the cytosol and mitochondrial matrix. **(A)** Scheme representing targeting of MICU1^1– 140^-GEMGeCO1, CVsue-GEMGeCO1, and ROMO1-GEMGeCO1 Ca^2+^ indicators within specific sub-mitochondrial regions. Dark gray region indicates intermembrane space (IMS) and light gray area the cristae lumen (CL). Simultaneous Ca^2+^ imaging of MICU1^1– 140^-GEMGeCO1 **(B,C)**, CVsue-GEMGeCO1 **(D,E)** and ROMO1-GEMGeCO1 **(F,G)** (green curves) with either CARGeCO1 (blue curve; **B,D,F)** or mt-CARGeCO1 (red curve; **(C,E,G)** co-expressing single HeLa cells treated with 100 μM histamine in the absence of extracellular Ca^2+^ and upon subsequent store-operated Ca^2+^ entry. Simultaneous measurements allow kinetical comparisons of the Ca^2+^ signals in the various (sub-)compartments.

The distinct spectral properties of the green and red GeCO1 biosensors allow simultaneous Ca^2+^ recordings in two distinct (sub-)compartments. First, we tested the suitability of the biosensors for simultaneous measurements by co-expressing MICU1^1–140^-GEMGeCO1 with MICU1^1–140^-CARGeCO1 and alternately recorded the respective emission. Since both biosensors exhibit a similar k_*D*_ for Ca^2+^, i.e., 340 nM for GEMGeCO1 (Zhao et al., 2011) and 490 nM for CARGeCO1 (Wu et al., 2013), simultaneous measurements resulted in almost analog Ca^2+^ traces in both channels even at randomly occurring oscillatory Ca^2+^ spikes in one given HeLa cell (Supplementary Figure S4). These results demonstrate that both, the red and green biosensor retain their full functionality even in sub-mitochondrial regions and further demonstrate the suitability of these constructs to image Ca^2+^ in a multi-chromatic manner. Accordingly, we co-expressed each sub-mitochondrial targeted GEM-GeCO1 with either the cytosolic- (CARGeCO1) or the mitochondrial matrix-targeted (mt-CARGeCO1) red Ca^2+^ biosensor and imaged the responses upon histamine stimulation and store-operated Ca^2+^ entry within individual cells. Targeting of the various sensors is illustrated in Figure 3A and the respective sub-mitochondrial Ca^2+^ measurements simultaneously with cytosolic Ca^2+^ signals (CARGeCO1) are presented in Figures 3B,D,F. Furthermore, simultaneous measurements of the mitochondrial matrix Ca^2+^ with the newly targeted Ca^2+^ sensors for the IMS, and for both CL probes were shown in Figures 3C,E,G. Notably, the various sensors exhibited a distinct kinetics that further reflecting the individual Ca^2+^ signaling of the very sub-compartment of the mitochondria. Hence, co-imaging of IMS with cytosolic Ca^2+^ in a fast temporal sampling mode revealed almost simultaneous occurring Ca^2+^ increase (Supplementary Figures S5A,B), while mitochondrial matrix Ca^2+^ was clearly delayed upon histamine stimulation (Supplementary Figures S5C,D).

### Knockdown of MCU/EMRE Augments the Ca^2+^ Accumulation Within the Mitochondrial Cristae

**FIGURE 4:**
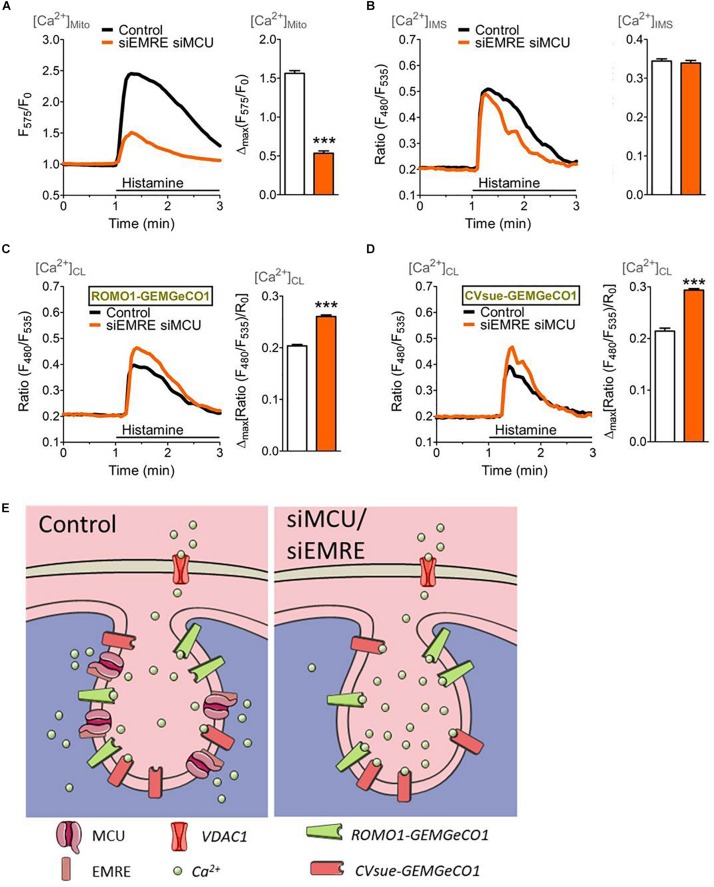
Mitochondrial (sub-)compartmental Ca^2+^ signaling upon knockdown of the pore-forming units of the mitochondrial Ca^2+^ uniporter complex, MCU and EMRE. HeLa cells were co-transfected with GeCO1 Ca^2+^ sensors either targeted in the mitochondrial matrix **(A)**, the IMS and **(B)** the CL targeted with either CVsue **(C)** or ROMO1 **(D)**, and specific siRNAs against EMRE and MCU (siEMRE siMCU, orange curves and bars) or scrambled control siRNAs (Control, black curves, and white bars). Cells were stimulated with 100 μM histamine in the absence of extracellular Ca^2+^. **(A)** Average mitochondrial Ca^2+^ signals over time measured with mt-CARGeCO1 (**left panel**) and statistical evaluation (**right panel**) of maximal Δ intensities upon siEMRE siMCU (*n* = 7) vs. Control (*n* = 8). **(B)** Average [Ca^2+^]_*IMS*_ curves of IMS-GEMGeCO1 expressing cells **(left panel**) and bars (**right panel**) representing maximal Δ ratios in Control (*n* = 9) and siEMRE siMCU (*n* = 8). **(C)** Average Ca^2+^ signals within the CL recorded from CVsue-GEMGeCO1 expressing HeLa cells (**left panel**) and statistical analysis (**right panel**) of Control (*n* = 9) in comparison to siEMRE siMCU (*n* = 9). **(D)** Average [Ca^2+^]_*CL*_ traces of ROMO1-GEMGeCO1 expressing cells (**left panel**) and bars (**right panel**) representing maximal ratiometric fluorescence changes for Control (*n* = 11) and siEMRE siMCU (*n* = 10). ^∗∗∗^*P* < 0.001 vs. Control. **(E)** Illustration of cristae localized Ca^2+^ indicators (ROMO1-GEMGeCO1 or CVsue-GEMGeCO1) sensing Ca^2+^ upon IP_3_-mediated Ca^2+^ mobilization within the cristae lumen under control (**left panel**) or under ablation of mitochondrial Ca^2+^ uptake proteins (siEMRE siMCU, **right panel**).

To verify the effect of an inhibition of mitochondrial Ca^2+^ uptake on the IMS and CL Ca^2+^ signals, the expression of MCU and its positive key regulator EMRE (Baradaran et al., 2018) were reduced by transfection with the respective siRNAs. Knockdown of MCU and EMRE resulted in an effective decrease of mitochondrial Ca^2+^ uptake upon intracellular Ca^2+^ mobilization (Figure 4A), while the respective Ca^2+^ signal in the IMS remained unaffected (Figure 4B). However, ablation of MCU and EMRE yielded enhanced Ca^2+^ signals in the CL measured with CVsue-GEMGeCO1 (Figure 4C) as well as with ROMO1-GEMGeCO1 (Figure 4D) upon stimulation with histamine. These results point to a specific Ca^2+^ accumulation that occurs especially within the CL under conditions where mitochondrial Ca^2+^ uptake is prevented by the knockdown of MCU/EMRE (Figure 4E).

### Knockdown of VDAC1 Impedes the Ca^2+^ Transfer Through the OMM

**FIGURE 5:**
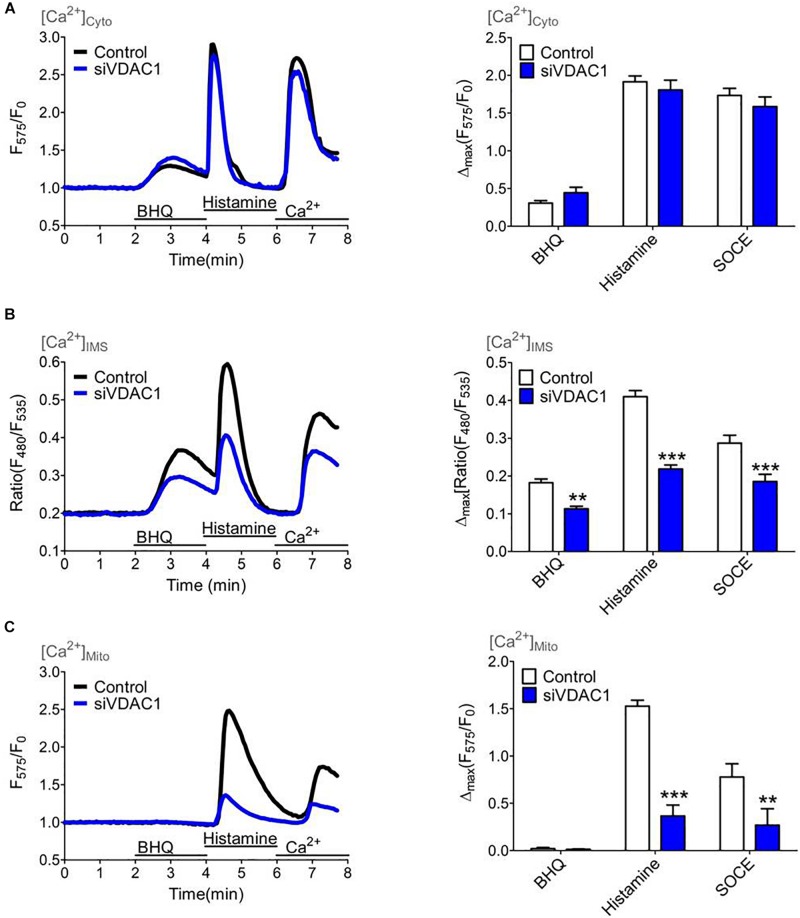
Mitochondrial (sub-)compartmental Ca^2+^ signaling upon knockdown of VDAC1 that controls the Ca^2+^ transfer through the outer mitochondrial membrane (OMM). HeLa cells were treated with either a siRNA against VDAC1 (siVDAC1, blue curves and bars) or a scrambled Control siRNA (Control, black curves and white bars) and Ca^2+^ imaging **(A–C)** was performed over time by consecutively application of 15 μM BHQ and 100 μM histamine in the absence of extracellular Ca^2+^ following a readdition of extracellular Ca^2+^ to highlight store-operated Ca^2+^ entry. **(A)** Average cytosolic Ca^2+^ curves (**left panel**) and statistical analysis for Control (*n* = 11) and for siVDAC1 (*n* = 11) of maximal Ca^2+^ elevations during treatment with either 15 μM BHQ, 100 μM histamine or re-addition of 2 mM Ca^2+^ (SOCE) (**right panel**). **(B)** Average curves of [Ca^2+^]_*IMS*_ (**left panel**) and statistical evaluation of maximal Δ ratio signals (**right panel**) for Control (*n* = 19) versus siVDAC1 (*n* = 19). **(C)** Average curves reflecting mitochondrial Ca^2+^ signals (**left panel**) and bar graph (**right panel**) showing maximal normalized Δ intensities for Control (*n* = 17) and siVDAC1 (*n* = 10). ^∗∗^*P* < 0.01 and ^∗∗∗^*P* < 0.001 vs. Control.

Several studies have shown that VDAC1 serves as a gate for Ca^2+^ ions within the OMM (De Stefani et al., 2011). Hence, we performed siRNA mediated knockdown of VDAC1 that was validated on the mRNA level by qRT-PCR (Supplementary Figure S6). To monitor the Ca^2+^ transfer through the OMM we used the IMS targeted MICU1^1–140^-GEM-GeCO1 in comparison with cytosolic CARGeCO1 or mt-CARGeCO1 and established a protocol where we exposed the biosensors to either slow/low or fast/high ER Ca^2+^ release and store operated Ca^2+^ entry by the re-addition of extracellular Ca^2+^ after excessive ER store depletion. Notably, in comparison to the IP_3_-mediated ER Ca^2+^ release the SERCA inhibitor BHQ mediates a lower Ca^2+^ rise in the cytosol (Figure 5A) and the IMS (Figure 5B) that is marginally entering the mitochondrial matrix (Figure 5C). Knockdown of VDAC1 did not affect [Ca^2+^]_*Cyto*_ (Figure 5A). In contrast, depletion of VDAC1 resulted in a reduced Ca^2+^ elevation in the IMS (Figure 5B) and, much more pronounced, in the mitochondrial matrix (Figure 5C) under all conditions of cytosolic Ca^2+^ elevations.

## Discussion

In this study we identified specific sequences that allow the distinct targeting of genetically encoded Ca^2+^ sensors for (simultaneous) sub-mitochondrial Ca^2+^ measurements. We aimed to establish such biosensors for sub-mitochondrial Ca^2+^ measurements in order to allow the examination of spatial, sub-organellar Ca^2+^ kinetics that will foster our attempts in investigating mitochondrial Ca^2+^ homeostasis. Besides the identification of suitable signal sequences, targeting was assessed using super-resolution structural-illumination microscopy (SIM), and a comparative characterization of the very Ca^2+^ sensitivity was performed. Hence, our work also provides initial (simultaneous) measurements where we applied the new sub-mitochondrial Ca^2+^ sensors for analyses of the Ca^2+^ kinetics upon given intracellular Ca^2+^ signals and, thus, tested the suitability of these new Ca^2+^ sensors to reveal distinct sub-mitochondrial Ca^2+^ signals under various conditions of altered mitochondrial Ca^2+^ signaling.

So far, only few attempts to target fluorescent indicators to these very sub-mitochondrial regions were successful. In an early study, the cDNA of glycerolphosphate dehydrogenase (GPD) was fused to HA1-tagged aequorin to study [Ca^2+^]_*IMS*_ (Pinton et al., 1998) or [H^+^]_*IMS*_ (Porcelli et al., 2005), but at that time the authors did not determine the exact sub-mitochondrial localization. In a more recent work specific IMS- or CL-targeted pH sensors were introduced and employed on the investigation of mitoflashes within various mitochondrial regions (Rosselin et al., 2017). However, in the present work we seek for signaling sequences from proteins that are directly involved in Ca^2+^ ion signaling and of those the localization has been previously undoubtfully proven. We pictured the localization sequence of MICU1 as very promising approach as it not only includes the important polybasic region between the amino acids 99 to 110. By fusing either the ratiometric green GEMGeCO1 (Zhao et al., 2011) or the intensiometric red CARGeCO1 (Wu et al., 2013) with the C-terminal side of amino acids 1 to 140 from MICU1 we achieved an excellent targeting of the probes exclusively in the IMS but not the CL or mitochondrial matrix. Our approach for targeting Ca^2+^ sensors exclusively into the cristae was successful when using the recently identified proteins referred to as complex V subunit e (CVsue) and reactive oxygen species modulator 1 (ROMO1) that has been shown to localize exclusively in the CL (Norton et al., 2014; Rieger et al., 2014; Cogliati et al., 2016). Our findings show that displacing the ROS-sensitive cysteines of ROMO1 to serines at positions 15, 27, 42, and 79, and C-terminal fusion with the N-terminus of the Ca^2+^ sensors did not affect the proteins distinct CL localization, thus, this approach successfully provided us a strategy to localize (Ca^2+^) sensors exclusively to the CL.

In course of the functional testing of the newly targeted GEMGeCO1 Ca^2+^ sensors the ratiometric behavior upon changes in spatial Ca^2+^ was verified for all localizations. However, the ratio amplitude within the IMS was found to be approximately 1.5-fold higher than that obtained in the CL. One explanation for such reduced amplitude would be either a reduced Ca^2+^ accumulation within the CL. Alternatively, the fluorescence dynamic of GEMGeCO1 might be most likely reduced in the CL because of the acidic environment (Rosselin et al., 2017). By combining distinctively targeted ratiometric green fluorescent ratiometric GEMGeCO1 (Zhao et al., 2011) with differently targeted red intensiometric CARGeCO1 (Wu et al., 2013) we established simultaneous measurements of the Ca^2+^ signals of two distinct sub-mitochondrial compartments. The kinetical analysis of the various Ca^2+^ signals upon intracellular Ca^2+^ release revealed a tight and almost instant coupling between the cytosol and the intermembrane space, thus, indicating that Ca^2+^ transfer through the OMM is very rapid and does not represent a speed-limiting process. Such process is known for the mitochondrial Ca^2+^ uniporter complex that is essentially activated upon Ca^2+^-triggered rearrangement of MICU1 that also requires rather high Ca^2+^ concentrations (>4 μM) to get initiated (Waldeck-Weiermair et al., 2015). However, the kinetics of mitochondrial Ca^2+^ uptake complex (MCUC) is much lower than that in any other mitochondrial sub-compartment, thus indicated some further processes necessary for MCUC activation. This assumption is further supported by the findings that upon a slow Ca^2+^ release by SERCA inhibition or when Ca^2+^ flux through the OMM is reduced by knockdown of VDAC1, no or only very small matrix Ca^2+^ signals occur despite the Ca^2+^ signals in the IMS is much less affected. Our findings that the prevention of MCUC by knockdown of MCU and EMRE did not affect the Ca^2+^ signal in the intermembrane space but considerable increased Ca^2+^ accumulation in the CL point to an active contribution of the cristae to mitochondrial Ca^2+^ homeostasis.

Altogether, in the present work we have successfully targeted well-known Ca^2+^ sensors to sub-compartments of the mitochondria and established simultaneous measurements of two distinct locations. Hence, the newly targeted Ca^2+^ sensors described herein have been approved to be suitable for analyses of sub-mitochondrial Ca^2+^ signaling and mitochondrial Ca^2+^ homeostasis, thus, providing suitable tools to discriminate between malfunction of OMM and/or IMM Ca^2+^ import e.g., during generation of neurodegenerative disease and will foster our understanding on mitochondrial Ca^2+^ homeostasis in future work.

## DATA AVAILABILITY STATEMENT

The raw data supporting the conclusions of this manuscript will be made available by the authors, without undue reservation, to any qualified researcher.

## Author Contributions

JR-M, MD, CK, EE, HB, SB, and MW-W performed design and construction of sensors. JR-M, CK, and MW-W performed cloning and delivered plasmid material. MW-W and GZ performed imaging experiments and data analysis. CM-S performed qRT-PCR experiments including analysis. BG performed super-resolution SIM experiments and analysis. MW-W together with RM, BG, and WG supervised the project, performed data interpretation and wrote the manuscript. All authors discussed the results and commented on the manuscript at all stages.

## Conflict of Interest

The authors declare that the research was conducted in the absence of any commercial or financial relationships that could be construed as a potential conflict of interest.
