# Supplementary material for: Development and Application of Sub-Mitochondrial Targeted Ca2 + Biosensors
Source: Front Cell Neurosci. 2019 Oct 4;13:449. doi: 10.3389/fncel.2019.00449 (PMC6788349; doi:10.3389/fncel.2019.00449)
Supplement: Supplementary file 1 [file Data_Sheet_1.docx]

**Development and application of sub-mitochondrial targeted Ca^2+^ biosensors**

**Markus Waldeck-Weiermair^1,^*, Benjamin Gottschalk^1^, Corina T. Madreiter-Sokolowski^1,2^, Jeta Ramadani-Muja^1^, Gabriela Ziomek^1^, Christiane Klec^1,3^, Sandra Burgstaller^1^, Helmut Bischof^1^, Maria R. Depaoli^1^, Emrah Eroglu^4^, Roland Malli^1,5^, Wolfgang F. Graier^1,5^**

^1^ Molecular Biology and Biochemistry, Gottfried Schatz Research Center, Medical University of Graz, Neue Stiftingtalstraße 6/6, 8010 Graz, Austria

^2^ Energy Metabolism Laboratory, Institute of Translational Medicine, D-HEST, Swiss Federal Institute of Technology (ETH), Schorenstrasse 16, 8603 Schwerzenbach, Zurich, Switzerland

^3^ Division of Oncology, Department of Internal Medicine, Medical University of Graz, Stiftingtalstraße 24/1/III, 8036 Graz, Austria

^4^ Brigham and Women's Hospital, Department of Medicine, Harvard Medical School, 75 Francis Street, MA 02115, Boston, USA

^5^ BioTechMed Graz, Austria

Supplementary Material

## Supplementary Table

| ***Name (with N-terminally fused targeting sequence)*** | ***Localization*** | ***Ca^2+^ indicator*** | ***K_d_ for Ca^2+^***  ***(Hill coeff.)*** | ***pK_a_*** | ***Reference*** |
| --- | --- | --- | --- | --- | --- |
| MICU1^1-140^-GEMGeCO1 | IMS | GEM-GeCO1 | 340 nM  (2,94) | 6,16 | (Zhao et al., 2011) |
| CVsue-GEMGeCO1 | CL |  |  |  |  |
| ROMO1-GEMGeCO1 | CL |  |  |  |  |
| MICU1^1-140^-CARGeCO1 | IMS | CAR-GeCO1 | 490 nM  (2,01) | 5,74 | (Wu et al., 2013) |
| ROMO1-CARGeCO1 | CL |  |  |  |  |

**Supplementary Table 1 |** Overview and properties of generated sub-mitochondrial Ca^2+^ biosensors.

## Supplementary Figures


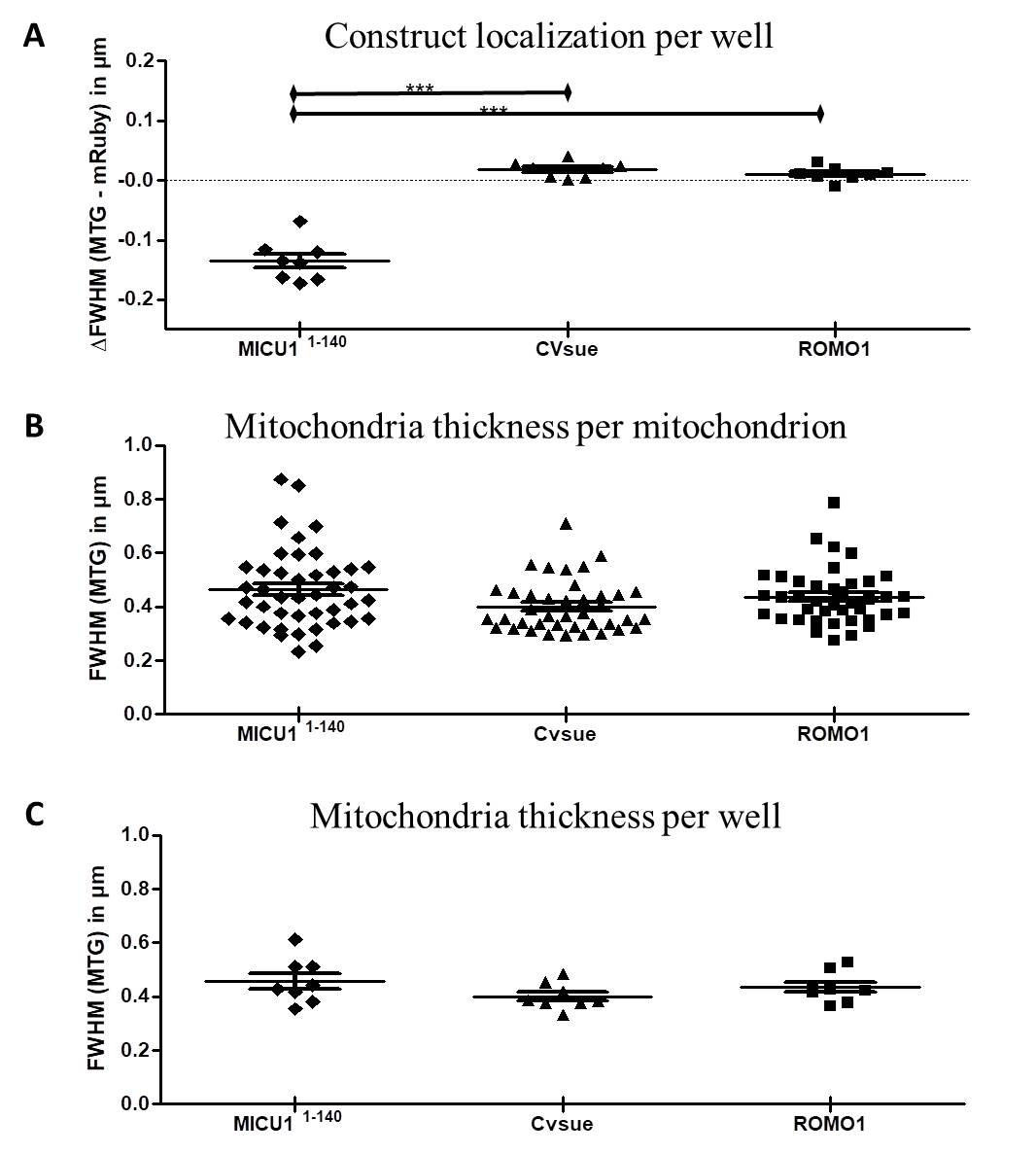


**Supplementary Figure 1 |** Dual-color structural illumination (SIM) analysis of sub-mitochondrial constructs**.** Complementary analysis of MTG loaded HeLa cells expressing either MICU1^1-140^-, CVsue- or ROMO1-mRuby presented in Figure 1 (**A**) Statistical evaluation of ΔFWHM values calculated per well; ***P<0,001 and (**B**) FWHW_MTG_ indicating mitochondria thickness per mitochondrion or (**C**) per well.

**
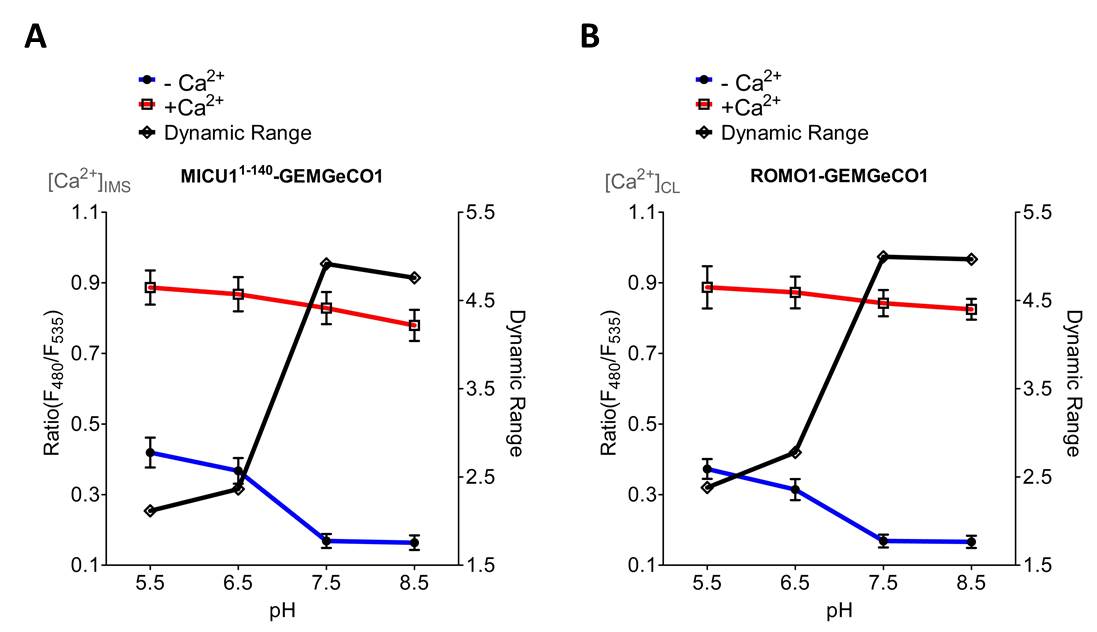
**

**Supplementary Figure 2 |** Emission ratio and dynamic range (black curves) of IMS or CL localized GEMGeCO1 tested at pH ranging from 5.5 to 8.5 in the presence (red curves) or absence of Ca^2+^ (blue curves) in (**A**) MICU1^1-140^-GEMGeCO1 (n=3) or (**B**) ROMO1-GEMGeCO1 (n=3) expressing HeLa cells.

**
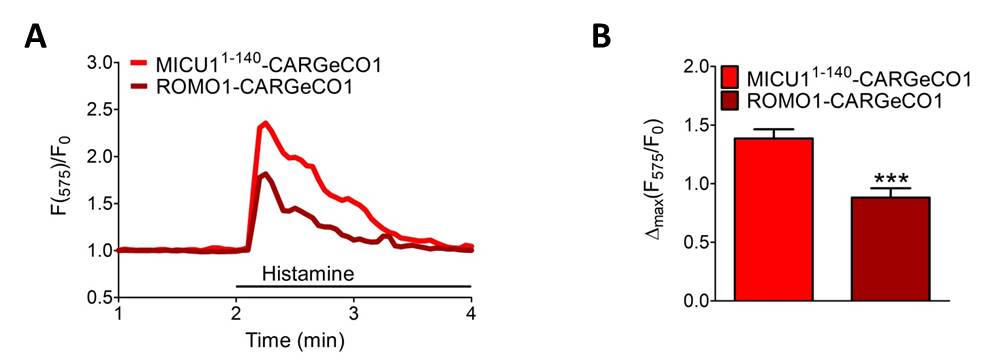
**

**Supplementary Figure 3 |** Performance of CAR-GeCO1 Ca^2+^ indicators fused with sub-mitochondrial targeting sequences. (**A**) Normalized average curves of Ca^2+^ measurements in HeLa cells expressing either MICU1^1-140^-CARGeCO1 (*left panel*, red curve) or ROMO1-CARGeCO1 (*left panel*, dark red curve), respectively stimulated with histamine in a nominal Ca^2+^-free environment. (**B**) Statistical evaluation of maximal Ca^2+^ elevation in [Ca^2+^]_IMS_ (n=11, *right panel*, red column), and [Ca^2+^]_CL_ (n=10, *right panel*, dark red column). ***P<0,001.

**
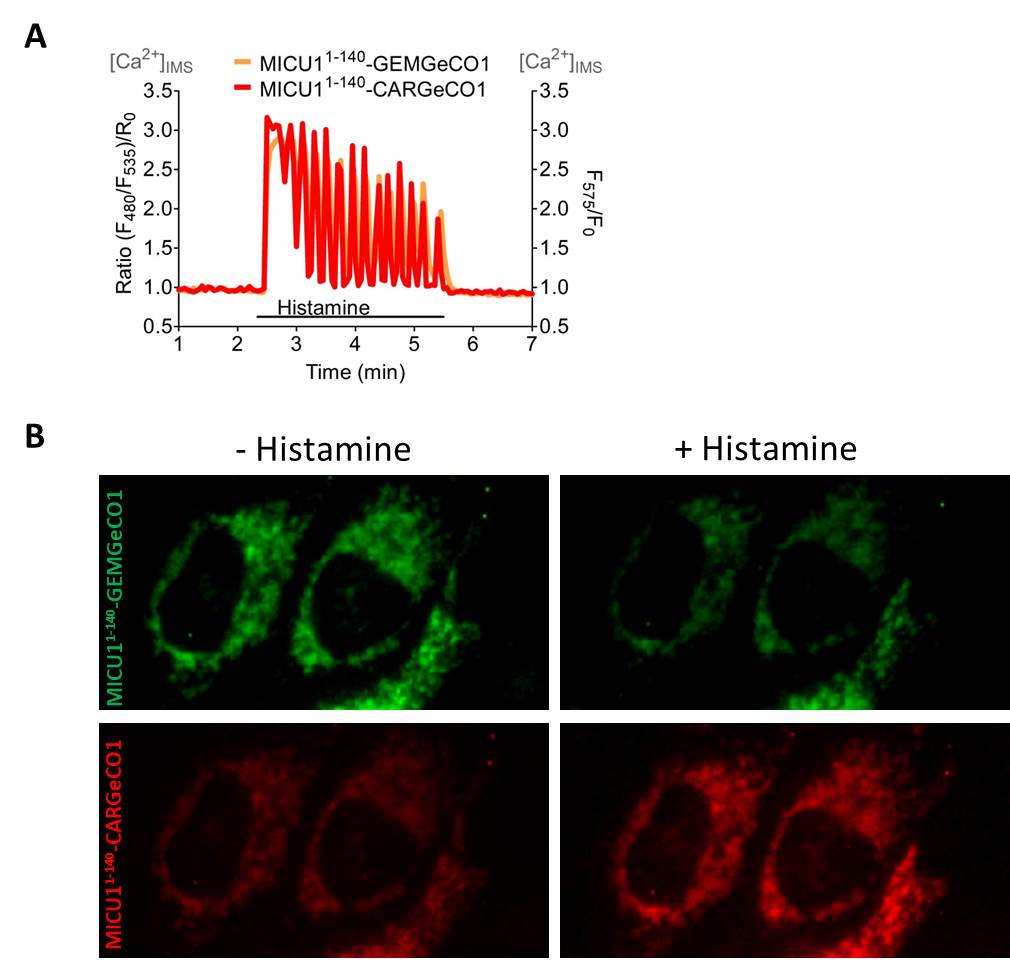
**

**Supplementary Figure 4 |** Simultaneous measurement of red and green Ca^2+^ indicators within the IMS. (**A**) Normalized fluorescence signals of IMS-GEMGeCO1 (green curve) and IMS-CARGeCO1 (red curve) upon 100 µM histamine stimulation in 0CaNa buffer. (**B**) Images as simultaneously recorded on the wide field microscope of either IMS-GEMGeCO1 (YFP channel, *upper panels*) or IMS-CARGeCO1 (RFP channel, *lower panels*) at resting Ca^2+^ levels (*left panels*) and at high Ca^2+^ levels (*right panels*) upon histamine treatment. Images of either GEMGeCO1 or CARGeCO1 have the same brightness and contrast settings.

**
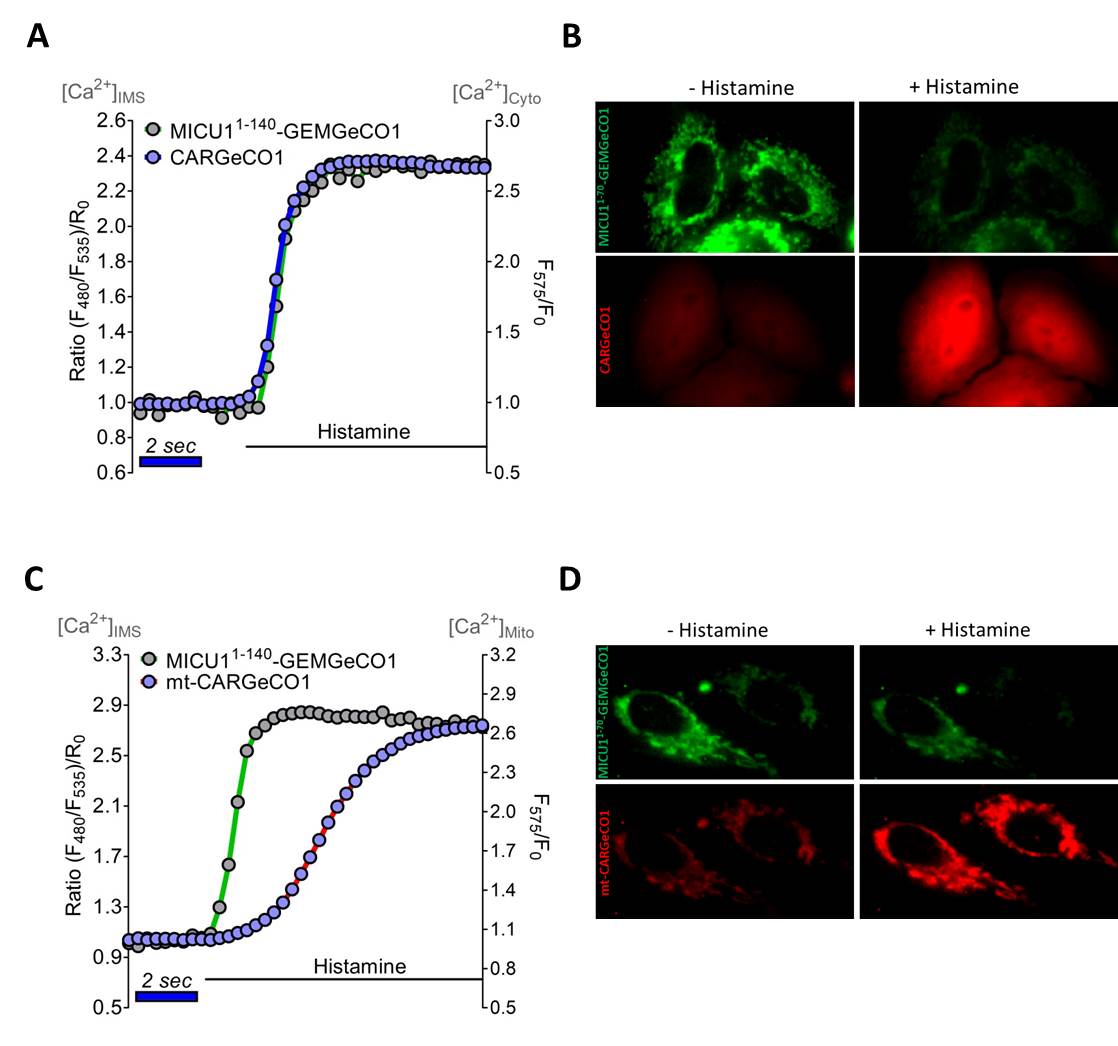
**

**Supplementary Figure 5 |** Dual recordings of IMS with either cytosolic or mitochondrial Ca^2+^ imaging. (**A**) Simultaneous Ca^2+^ traces of [Ca^2+^]_Cyto_ (blue curve) and [Ca^2+^]_IMS_ (green curve) both recorded within 300 ms intervals. (**B**) Acquired wide field images of MICU1^1-140^-GEMGeCO1 (YFP channel, *upper panels*) and cytosolic CARGeCO1 (RFP channel, *lower panels*) co-expressing HeLa cells before (*left panels*) and during stimulation with 100 µM histamine (*right panels*). (**C**) Dual ultrafast Ca^2+^ imaging of MICU1^1-140^-GEMGeCO1 (green curve) and mt-CARGeCO1 (red curve) within one co-expressing HeLa cell treated with 100 µM histamine (**D**) Dual Ca^2+^ recordings of MICU1^1-140^-GEMGeCO1 at 535 nm (*upper panels*) and mt-CARGeCO1 at 600 nm emission (*lower panels*) in HeLa cells before (*left panels*) or during of histamine treatment (*right panels*). Images of either GEMGeCO1 or CARGeCO1 have the same brightness and contrast settings.


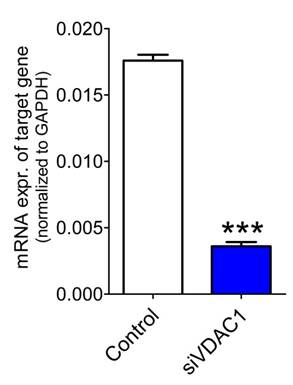


**Supplementary Figure 6 |** **Validation of siRNA mediated knockdown of VDAC1.** Validation of siRNA against VDAC1 transfected HeLa cells by qRT-PCR reveals ~ 80 % VDAC1 knockdown efficiency (siVDAC1, blue column) versus scrambled Control siRNA transfected cells (Control, white column), ***P<0,001 vs. Control.

**References**

Wu, J., Liu, L., Matsuda, T., Zhao, Y., Rebane, A., Drobizhev, M., et al. (2013). Improved orange and red Ca2+indicators and photophysical considerations for optogenetic applications. *ACS Chem. Neurosci.* 4, 963–972. doi:10.1021/cn400012b.

Zhao, Y., Araki, S., Wu, J., Teramoto, T., Chang, Y. F., Nakano, M., et al. (2011). An expanded palette of genetically encoded Ca2+ indicators. *Science (80-. ).* 333, 1888–1891. doi:10.1126/science.1208592.
